# Servant Leadership, Organisational Citizenship Behavior and Creativity: The Mediating Role of Team-Member Exchange

**DOI:** 10.5334/pb.326

**Published:** 2016-10-19

**Authors:** Winifrida Malingumu, Jeroen Stouten, Martin Euwema, Emmanuel Babyegeya

**Affiliations:** 1 KULeuven, BE; 2 The Open University of Tanzania, TZ

**Keywords:** Servant Leadership, OCB, Creativity, TMX

## Abstract

Using a multi-source field study design with 184 unique triads of employees-supervisor dyads, this paper examines whether servant leaders install a serving attitude among employees. That is, servant leaders aim to encourage employees to take responsibility, to cooperate and to create high quality interactions with each other (team-member exchange; TMX). We hypothesise that servant leadership will have an influence on Organisational Citizenship Behavior (OCB) and creativity through team-member exchange. Two facets of OCB are distinguished: organisational citizenship behaviour towards individuals (OCBI), on the one hand, and taking up extra tasks that benefit the organisation (OCBO), on the other hand. The results show that servant leadership is positively related to team-member exchange, and that team-member exchange is positively related to OCBI, OCBO and creativity. The bootstrapping estimates indicated significant indirect effects of servant leadership on the three target variables through team-member exchange. The study’s findings add to the body of literature on servant leadership, OCB and creativity at the workplace, and underline the importance of creating favourable working conditions that foster positive and high quality team-member exchange. This study also broadens our understanding on the importance of co-workers on the relation between servant leadership and organizational citizenship behavior (OCB) and creativity.

## Publisher's Note

This article was originally published with the incorrect pagination. This error has now been fixed.

## Article

Organisations heavily rely on employees to support each other, especially now when organisations operate in an economically unpredictable context (Maxel, 2013) and face competitive demands to meet their stakeholders’desires. Gaining a stronger competitive advantage requires leadership to encourage employees to go beyond mere task demands and going beyond the status quo by engaging employees in finding creative solutions for existing problems (Hoegl & Parboteeah, 2007). Such a focus requires leadership capable of recognising, utilising and developing employees’ potential and encouraging them to go the extra mile (Liden, Wayne, Zhao, & Handerson, 2008). Servant leadership brings out the best in people’s potential abilities (Greenleaf, 1977). Moreover, servant leadership is aimed at fostering a long-term relationship with employees and focuses on recognising the needs, goals, and abilities of employees to let them grow in the best possible way in the organisation and their career (Greenleaf, 1977; Liden et al., 2008).

**Figure 1:**
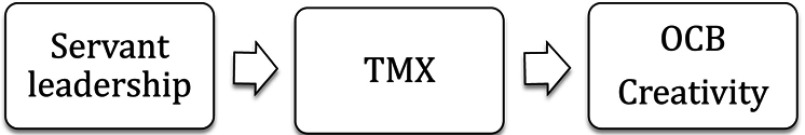
Proposed model on the Relationship between Servant Leadership, Team-Member Exchange, OCB and Creativity.

Indeed, leadership plays an integral role in the process of invigorating employees’ creativity (Neurbert, Kacmar, Carlson, Chonko, & Roberts, 2008) and discretionary behavior by taking up extra tasks or helping co-workers (Organisation Citizenship Behavior: OCB). Amabile and colleagues (1996) argue that creativity is central for organisations to attain competitive advantage. In this regard, creativity can be defined as the process of imagining and creating new ideas which are useful in improving services, processes and procedures in organisations (Amabile, Conti, Coon, Lanzenby, & Herron, 1996). Some early evidence was offered by Yoshida, Sendjaya, Hirst, and Cooper (2013) who showed that servant leadership affects employees’ creativity and team innovation. Moreover, Organisational Citizenship Behaviour (OCB) defined as the act in which employees are ready and willing to go beyond the call of duty (Organ, 1988), is also an important asset for organisations to successfully operate. Vondey (2010) indeed found that servant leadership predicts Organizational Citizenship Behavior (OCB) Figure 1.

An important premise of both creativity and OCB is that employees take responsibility in cooperating with each other to reach incremental value for the team and organization. Servant leaders have been argued to potentially create a servant mind-set in employees so that they are encouraged to help colleagues and the organization and work successfully together to find creative solutions to tackle specific work problems. The literature on servant leadership indeed contends that servant leaders promote selfless cooperation so that team members in close interaction with each other share valuable information and have regular high quality exchanges. Even though this has been argued, it has not been empirically tested. That is, little evidence (if any) is available to show that servant leadership could help employees to engage in such positive exchanges among workers (TMX) and as a result influences employees to serve colleagues and the organization. Seers, Petty and Cashman (1995) define TMX as team member’s perceptions of the quality of “reciprocity between a member and his team with respect to the member’s contribution of ideas feedback and assistance to others and, in turn, the member’s receipt of information, help and recognition from other team members” (p. 21). This study aligns with a call for more research to establish an understanding on how to achieve high quality group relations and cooperation (Leary, 2007). Because OCB and creativity are competitive assets for organizations and they align with serving each other and the organization, we contend that servant leadership can set a stage for extra role behavior and encourages the creative process by building such high quality exchanges between employees (TMX). Hence, we tested an important underlying premise in the servant leadership literature that it emphasizes taking up responsibility and create high quality relationship among workers.

This paper contributes to servant leadership, OCB and the creativity literature in three different ways. First, it examined the relationships between servant leadership and OCB both aimed at organizations and individuals (OCBO and OCBI). Moreover, it links servant leadership and creativity. Servant leadership has the underlying premise that it helps employees to develop their potential, this should hence also translate in encouraging employees to go beyond mere task execution and find ways to look at things from a different perspective, take in other sources or skills; hence, being more creative in their jobs, an area in the servant leadership literature that is yet unexplored. Secondly, we examined the relationship between servant leadership and TMX. We contend that servant leaders create value for the team because servant leaders focus on emotional healing, as such creating value for the community and empowering behaviors (Liden et al., 2008), thereby fostering the development of a positive high quality TMX. We aimed to add to the servant leadership literature by focusing on TMX as an underlying mechanism for the association between servant leadership and OCB and creativity. Through servant leadership exchanges among team members are fostered and team members develop a serving attitude. Moreover, we also add to the creativity literature by introducing servant leadership and TMX as important mechanisms to enhance the creative process. We examine the relationship between TMX and improved OCB and creativity, pointing to TMX as a contextual variable. That is, through TMX, employees show elevated levels of interpersonal helping behaviors, as well as helping behaviors targeted to the organization as a whole (by e.g. defending the organization when needed). Throughout our paper we refer to the two forms of OCB namely OCBO and OCBI. We propose that effective and quality TMX is vital in the process of attaining OCB and creativity, which is an overlooked area of research that connects leadership, OCB and creativity.

### Servant Leadership

Liden and colleagues (2008) distinguished between seven dimensions of servant leadership: emotional healing, creating value for the community, conceptual skills, empowering, helping subordinates grow and succeed, putting subordinates first, and behaving ethically. Servant leadership initiates a conducive working environment by serving others and by putting surbodinates’ interests first, and focusing on employees’own development and growth (Hu & Liden., 2011; Kark, & Carmeli, 2009; Liden et al., 2008; Schaubroeck, Sendjaya, Sarros, & Santora, 2008; Van Dierendonck, 2011). Indeed, when servant leaders encourage employees’ growth they are meant to become servant leaders themselves (Greenleaf, 1970; Liden et al., 2008). Servant leadership also creates values for the community as it focuses on service provision within and beyond the organisation (Van Dierendonck, 2011). Additionally, servant leadership goes beyond organizations by serving multiple stakeholders, including employees, their community, and society at large (Liden et al., 2008). Servant leaders do not only emphasise integrity and the creation of long-term relationships with employees (Liden et al., 2008; Stone, Russell, & Patterson, 2004) but also strive to empower their followers by integrating their ideas in the decision-making process. This creates openness and a sense of communal sharing and psychological safety, trust and fairness in the working context (Hu et al., 2011; Kark et al., 2009; Liden et al., 2008; Schaubroeck et al., 2011; Sendjaya, Saros, & Santora, 2008; Van Dierendonck, 2011), thus leading to a strong high-quality dyadic interpersonal relationship (Ferris, Liden, Munyon, Summers, Basik, & Buckley, 2009). Indeed, this makes employees feel free and more autonomous in their decision-making (Ferch, 2005). Above all, servant leadership accomplishes what is promised and as a result is seen as more thoughtful, trustworthy, and reliable (Liden et al., 2008; Page & Wong, 2000; Stone et al., 2004).

Servant leadership is unique in that it contains incremental validity beyond and above leader member exchange and transformational leadership (Liden et al., 2008). Moreover, servant leadership has been credited with characteristics which are less expressed in other constructs such as the focus on serving followers for their own growth by forming long-term relationships and inspiring them to become servant leaders themselves (Greenleaf, 1970; Liden et al., 2008), as well as the concern for multiple stakeholders (Walumbwa, Avolio, Gardner, Wernsing, & Peterson, 2008).

### Servant Leadership and Team-Member Exchange

Leader-follower relations can often be understood as a social exchange in which both supervisors and followers aim to balance costs and benefits (Homans, 1961). Social exchange is represented as an implicit co-ordination of behaviour. Social exchange theory clarifies that positive exchanges are reciprocal and are developed through social interaction experienced between two individuals (Blau, 1964); as they positively interact with each other and accumulate experiences, both being concerned about each other’s welfare. Central to social exchange processes is the norm of reciprocity, obligating individuals to respond in kind to the resources and support received from others. Indeed, servant leaders provide such resources and support through creating a working environment in which participation is central (Rezaei, Salehi, Shafiei, & Sabet, 2012), establishing a communal culture (Giampetro-Meyer, Brown, Browne, & Kubasek, 1998), being communicative and supportive (Gimbel, 2001) as well as providing general care for employees. In this regard, servant leaders need to transcend self-interest, express genuine care and concern, and act in the best interest of their followers. By helping subordinates grow and putting their interests first, servant leaders seek to enhance a high quality exchange between all team members. Employees are, therefore, encouraged to trust and help each other and take ownership to do so by sharing valuable expertise and information. As such, servant leadership enables positive relationships to thrive among employees which allow them to exchange trustfully resources and ideas with each other, or put differently, to enhance team-member exchange (TMX).

TMX represents an individual’s overall perception of exchanges with other members of the work group; this exchange can vary in terms of the content and process of exchange. In case of low TMX, exchanges are limited to what is required for the completion of the task. High TMX, on the other hand, involves an exchange of resources and support that extends beyond what is necessary for task completion (Liden et al., 2000), which parallels with servant leaders’ focus on developing employees and putting subordinates first.

Therefore, we hypothesize:

H1: Servant leadership will be positively related to team-member exchange.

#### Team-Member Exchange and OCB

A positive exchange between team-members highlights the supportive relations that exist among employees. Drawing on social exchange theory (Blau, 1964), we argue that employees will reciprocate the positive exchange by being motivated to go the extra mile and taking up tasks that go beyond their regular tasks, that is OCB. OCB can be defined as an “individual behavior that is discretionary, not directly or explicitly recognised by the formal reward system and that in the aggregate promotes the effective functioning of the organisation” (Organ, 1988, p. 4). OCB can be distinguished between OCB towards colleagues and helping out co-workers (organisational citizenship behavior towards individuals), on the one hand, and towards taking up extra tasks that benefit the organisation (organisational citizenship behavior towards the organization), on the other hand. The little research that focused on the relation between TMX and OCB indeed indicates that TMX is positively related to OCB (Love & Forret, 2008) and also specifically to OCB related to helping (Kamdar & Van Dyne, 2007). We build on these findings by further distinguishing between OCB directed at the organization (OCBO) and targeted to helping co-workers (OCBI). We contend that the level and quality of exchange depends on how an employee perceives the exchange with the peers or group as a whole (Seers, 1989, p. 119). In this respect, high quality TMX signifies the existence of a well-established relationship that exists between employees. The existence of such a positive relationship catalyses employees’ positive feelings towards the organisation (OCBO) and towards co-workers (OCBI). Indeed, high-quality TMX builds a strong foundation and guidance for employees’ actions as it creates trust and strengthens relationships within the team and among individuals. In this regard, we argue that high TMX sets for a supportive and helping environment in which employees become willing to act and collaborate to achieve organisation as well as co-worker success.

We thus hypothesize that:

H2a: Team-member exchange will have a positive relationship with OCBI

H2b: Team-member exchange will have a positive relationship with OCBO

#### Team-Member Exchange and creativity

If employees supportively share valuable information and interact, they are able to create opportunities for new initiatives and the generation of new ideas in one’s work (cf. creativity). Creativity is dependent on the team exchange process. Indeed, research by Zhou and George (2001) found a significant positive relationship between the help, support and feedback from colleagues and creativity for dissatisfied employees. More recently, research by Muñoz-Duyague and Nieto (2012) found that high-quality TMX has a significant positive influence on employees’ creative behaviour. In the context of high quality TMX, an employee is willing to offer support and share information, resources, provide and receive feedback in a constructive way from and to other employees. Team members will also have an opportunity to leverage knowledge and skills from other team members (Hoegl & Wagner, 2005). This is primarily because employees feel that they are not alienated from the group (Seers, 1995; Jordan, Field, & Armenakis, 2002). Similarly, Amabile and Gryskiewicz (1989) and Sethia (1991) noted that collaboration efforts between colleagues are fundamental for the generation of creative ideas. TMX creates such positive exchanges and enables employees to think creatively.

Therefore, we hypothesize that:

H3: Team-member exchange will have a positive relationship with creativity

Servant leaders do not only encourage employees to develop their skills but also empower them to carry out their jobs in the way they feel is right and to make decisions regarding how best to execute the job. With the best interest in mind, servant leaders assist followers to achieve their full potential (Liden et al*.*, 2008; Lord, Brown, & Freiberg, 1999). Developing and empowering employees’ servant leadership accomplishes this feat by ensuring there is interpersonal acceptance, stewardship, humility and guiding subordinates (Van Dierendonck, 2011). Research has highlighted that once employees are empowered; they demonstrate confidence and can positively influence their working environment (Zhu, May, & Avolio, 2004). Such a situation contributes to the development of positive attitudes such as organisational citizenship behavior (Sendjaya, Saros, & Santora, 2008). Hence, servant leaders stimulate a high quality exchange among team members contributing to members taking initiatives and behavior acting upon this by exemplifying OCB.

Given that servant leadership is expected to positively relate to TMX, and that TMX is expected to relate to OCB, we argue that the indirect relationship between servant leadership and OCBI and OCBO through TMX is significant.

H4a: There is an indirect relationship between servant leadership and employees’ OCBI through TMX

H4b: There is an indirect relationship between servant leadership and employees’ OCBO through TMX

Shin and Zhou (2007) argued that the environment determines the extent to which employees are ready to contribute creatively. If employees experience support from colleagues, and they feel that the environment is a safe place to initiate new ideas, this makes employees ready to interact and exchange information with openness in the process of developing new ideas without fear of being condoned. Servant leadership can thus set the stage for positive and high TMX which, in turn, relates to creativity. We therefore hypothesise:

H5: There is an indirect relationship between servant leadership and employees’ creativity through TMX

### Method

#### Participants, Sample and Sampling Procedure

The study involved a total sample of 350 respondents consisting of employees, their co-workers and supervisors. To reduce the common method variance, we triangulated the data, that is, we collected data from the focal employees, co-workers and their supervisors, hence resulting into 188 unique triads (54% response rate). To form triads, the employees were matched with their co-workers and supervisors. Employees were from different organisations in Belgium drawn from the medical, human resource, food service, financial, insurance, retail service, manufacturing, government, and technology sectors. We sent an electronic link to the focal employees who were requested to send the link to their supervisors through e-mail together with an invitation to participate in the survey as well as to a co-worker who was familiar with their work. The researchers guaranteed anonymity and confidentiality in the data collected. Because of the triangulated nature of the study, we emphasised the significance of integrity and trustworthiness in filling out the survey instrument, that is, it was vital for employees and co-workers to fill their respective survey (correct survey) and not otherwise. We found no irregularities.

Supervisors constituted 51.5 percent females and were on average aged 45 years (*SD* = 9.73); they had worked on average for 15.96 years in their respective organisation (*SD* = 11.14). A majority of the supervisors had a Master’s degree (41.60%), 17.90% had a Bachelor’s degree and 13% had completed secondary school but 27.50% did not indicate their education level.

Employees were 53.8 percent females with an average age of 39.76 years (*SD* = 11.93) and an average tenure in their organisation of 12.18 years (*SD* = 11.19). In terms of education, 50.0 percent had a Master’s degree, 23.30 percent had a Bachelor’s degree, and 21.70 percent had only completed secondary education and five percent did not indicate their level of education.

Co-workers were 57 percent females with an average age of 37.10 years (*SD* = 10.72). On average, they had worked for 10.89 years in their organisation (*SD* = 10.35). In terms of education, 8.0 percent obtained a Master’s degree, 15.30 percent had a Bachelor’s degree, 29.0 percent had a high school degree and 19.1 percent completed secondary education, whereas 28.60 percent did not indicate their education level.

#### Measures

Measures for our study were completed by the research participants as follows: Servant leadership and Team-member exchange were assessed by focal employees, while OCBO and OCBI were assessed by co-workers and employees’ creativity was assessed by supervisors. All measures were scored on a 5 point likert scale (1 = strongly disagree, 5 = strongly agree).

**Servant Leadership:** Servant leadership was assessed by focal employees using the 28-item multidimensional measure of servant leadership developed by Liden et al. (2008). A sample item was “My manager makes my career development a priority.” Cronbach’s alpha was .92.

**Team-Member Exchange:** Team-member exchange was assessed by focal employees using a 9-item scale developed by Liden, Wayne and Sparrowe (2000). A sample item was: “My co-workers have asked for my advice in solving a job-related problem of theirs.” Cronbach’s alpha was .86.

**Organisation Citizenship Behaviour (OCB):** OCBO and OCBI were assessed by co-workers with items developed by Lee and Allen, (2002) and each contained eight items. Example items are “I demonstrate concern towards the image of the organization” (OCBO). Cronbach’s alpha was .90. And “I help others who have been absent” (OCBI). Cronbach’s alpha was .86.

***Creativity***: Employees’ creativity was assessed by supervisors using a four-item scale developed by Tierney, Farmer, and Graen (1999). A sample item was: “This employee seeks new ideas and ways to solve problems.” Cronbach’s alpha was .92.

### Results

Table 1 shows the means, standard deviations, and inter-correlations. Missing data were deleted list wise. Control for the demographic variables (i.e., age, sex, education and tenure) did not significantly change the results. Therefore, we followed Becker’s (2005) recommendation and we present our findings without the control variables.

**Table 1:** Mean, Standard Deviations, and Inter-correlations.

	*M*	*SD*	1	2	3	4	5

1. Creativity	3.31	.95					
2. OCBI	4.09	.59	.23**				
3. OCBO	3.94	.65	.37**	.56**			
4. Team-member exchange	4.00	.57	.17*	.25**	.16**		
5. Servant leadership	3.44	.55	.05	.09	.03	.32**	

N = 184, * Correlation is significant at *p* < .05, ** Correlation is significant at *p* < .01.

Table 2 presents the regression coefficients, standard errors and model summary results for hypotheses 1, 2a, 2b and 3). Hypothesis 1 predicted that servant leadership is positively related to team-member exchange. Results of a regression analysis showed that servant leadership had a positive relationship with team-member exchange (b = .33, *p* < .001). Hypothesis 1 was therefore confirmed. With regard to hypothesis 2a, we predicted that team-member exchange is positively related to OCBI. Regression analysis results showed that team-member exchange is indeed positively and significantly related to OCBI (b = .24, *p* < .001). For hypothesis 2b, we predicted that team-member exchange is positively related to OCBO. Results indeed showed that team-member exchange related positively and significantly to OCBO (b = .17, *p* < .03). For the third hypothesis, we predicted that team-member exchange has a positive relationship with creativity. Analyses indeed showed a positive significant relation (b = .28, *p* < .02).

**Table 2:** Regression Coefficients for the Relation between Team Member Exchange, Creativity, OCBI and OCBO.

Creativity	b	SE	*t*

Team Member Exchange	0.28	0.17	2.35*
OCBI			
Team Member Exchange	0.24	0.07	3.47**
OCBO			
Team Member Exchange	0.17	0.08	.03*

**Notes:** *p < .05, **p < .001, Model 1: *F*(1,187) = 5.52, *p* < .02, R-squared = .03; Model 2: *F*(1,182) = 12.06, *p* < .001, Model 3: *F*(1,182) = 4.54, *p* < .03, R-squared = .02.

Finally, we tested for the indirect effect of servant leadership on Creativity, OCBI and OCBO through TMX. First, we tested for the relations between TMX and the target variables creativity, OCBI and OCBO when controlling for servant leadership. Analyses of creativity showed a significant relation (b = .28; 95% CI [0.0325, 0.5275]), but the F-test for the model only was significant at *p* < .10. Results showed that team-member exchange is positively and significantly related to OCBI (b = .23; 95% CI [0.0930, 0.3834]). Finally, results showed that team-member exchange related positively and significantly to OCBO (b = .17; 95% CI [0.0095, 0.3401]), yet the F-test for the model did not reach significance (See Table 3).

**Table 3:** Regression Coefficients, Standard Errors and Model Summary for Servant Leadership, Team-Member Exchange, Creativity, OCBI and OCBO.

Model	Team-member exchange			Creativity		

**1**	**b**	**SE**	***p***	**b**	**SE**	***p***
	
Servant Leadership	.33	.25	< .01	.003	.13	.97
Team-Member Exchange	–	–	–	.28	.13	.03
	*R*^2^ = .10			*R*^2^ = .03		
	*F*(1,187 ) = 20.53, *p* = .001			*F*(2,186) = 2 .74, *p* = .07		
**2**					**OCBI**	
	
Servant leadership	.33	.08	< .01	.02	.09	.85
Team-Member Exchange	–	–	–	.28	.13	< .01
	R^2^ = .09			R^2^ = .06		
	*F*(1,182 ) = 18.81, *p* = .001			*F*(2,181) = 6.01, *p* = .003		
**3**					***OCBO***	
	
Servant leadership	.33	.08	< .001	–.02	.09	.84
Team-Member Exchange	–	–	–	.17	.08	.04
	R^2^ = .09			R^2^ = .03		
	*F*(1,182 ) = 18.81, *p* = .001			*F*(2,181) = 2.28, *p* = .11		

In order to test Hypotheses H4a, H4b and H5, we calculated the indirect effect with bootstrapping techniques by using PROCESS macros for SPSS (Hayes, 2013). Bootstrapping is preferred because it treats the sample as a population by re-sampling and replacing it several times (5000) and compute appropriate statistics for each sample (Hayes, 2013). The bootstrapping technique reduces the sampling distribution anomaly by calculating confidence intervals (Hayes, 2013). Our results confirmed that servant leadership relates to creativity, OCBO and OCBI through TMX. We could therefore confirm H4a, H4b, and H5 (see Table 4).

**Table 4:** Indirect Effect of Servant Leadership on Team Creativity, OCBI and OCBO.

***Indirect effect of Servant Leadership on Creativity***				

	**B**	**SE**	**ULC195%**	**ULC195%**

Team-member exchange	.09	.05	.0135	.2174

***Indirect effect of Servant Leadership on OCBI***				

Team-member exchange	.08	.03	.0255	.1569
***Indirect effect of Servant Leadership on OCBO***				

Team-member exchange	.06	.03	.004	.1386

### Discussion

This study addressed the relation of servant leadership and OCB and employee creativity by focusing on the roles that leaders play in encouraging an enhanced team-member exchange (TMX). In this study, we examined how servant leaders can create high quality and effective TMX to enhance OCB and creativity. Our results are congruent with the hypothesized model in which servant leadership sets the stage of TMX, which in turn relates to higher extra-role behavior (OCBI and OCBO) and employees’ willingness to engage in developing new ideas so to enable organisational effectiveness and functioning. These findings highlight that servant leaders have a positive influence on creating an environment in which employees among each other create high quality exchanges. Employees share information, interact more trustfully and cooperatively which, in turn, encourages them to go the extra mile for co-workers and the organization. Moreover, TMX encourages employees to engage in the creative process by looking for alternative ways and taking a different perspective in the way one’s job is organised.

### Theoretical Implications

Our study contributes to the literature on servant leadership, OCB, and creativity in three different ways. First, this research adds to the servant leadership literature by addressing servant leaders’ role in initiating team-member exchange in fostering OCB and creativity. Empirically, servant leadership has been linked to many positive outcomes. However, this study adds to the literature by showing that servant leadership creates a climate of learning and growing in one’s job which translates in high quality exchanges among employees.

Second, the research adds to the literature on the relationship between TMX and OCB (OCBO & OCBI) and creativity. With regard to OCB, our study results demonstrated that, TMX is significantly and positively related to OCB at the individual as well as the organisational level (OCBO & OCBI-hereafter we refer to these as simply OCB). This implies that high quality TMX triggers employees to engage in OCB. Even though empirical studies in this area are limited, this study is in concurrence with prior research which shows that high-quality team-member exchange boosts team members’ helping behaviour and their intention to share knowledge with each other (Kamdar et al., 2007; Liu, Keller, & Shih, 2011; Love et al., 2008). With regard to creativity, findings from the current study show that TMX is positively and significantly related to creativity. This suggests that high team-member exchange relates to employees’ level of openness and support from co-workers as well as to an increased engagement in developing new ideas. This is in agreement with previous studies indicating that the quality of employees’ relationships is positively related to a safe and positive interpersonal environment, which makes employees feel comfortable when interacting with co-workers (May, Gilson, & Harter, 2004; Tse & Dasborough, 2008). Indeed, if employees perceive the work context to be safe and they are given freedom, they will be ready to contribute new ideas without fear of being condoned (Zaheer & Zaheer, 2006). This finding corroborates with Dollard and Bakker (2010) and May et al, (2004) who contend that a positive working environment leads to high-quality TMX, which in turn facilitates the employees’ role-making process and engagement. In our study, this refers to the process of engaging in team creativity through various initiatives.

Third, based on social exchange theory, the indirect relationship between servant leadership and OCB as well as creativity though team-member exchange is significant. This implies that there is a need to consider conditions that favour the development of positive and high TMX, which in turn foster OCB and employees’ creativity. Our study is consistent with social exchange theory, which according to Blau (1964) states that trustworthy actions initiate a sense of ownership in employees and support for each other. Moreover, our study corroborates with previous research which acknowledged the importance of co-worker quality social relationship and put emphasis on social exchange networks in the workplace (Cole, Schaninger, & Harris, 2002, Chiaburu & Harrison, 2008; Liao, Yang, Wang, Drown, & Shi, 2013).

### Practical Implications

The study has a number of practical implications. In terms of the relationship between servant leadership and TMX, results show that servant leadership has the ability to create an environment that promotes positive feelings in employees about their fellows and their leaders, which is necessary for building positive and high quality reciprocal exchange relations. This finding implies that servant leadership enables the creation of a safe and trustworthy environment by encouraging, empowering and creating interdependence and predictability between employees within the organization. Such conditions enable the organization to develop and promote a service oriented culture with socialized power and sharing spirit among its members.

Second, in terms of the relationship between TMX, OCB and creativity, our results show that TMX positively related to OCB and creativity. These findings show that TMX is an important aspect in the relation with OCB and creativity. The finding implies that organizations could design organisational structures that support and strengthen employees’ relationships and reciprocal exchanges. Such structures will enable organizations to provide required resources and foster feedback among employees. This is not only important for creating employee value but also enables trust to flourish. The developed structures will also help in designing training for employees in various social exchange skills such as sharing information, knowledge, skills and innovations.

### Limitations and Future Research

Although we found evidence to support our hypotheses, our study is not without limitations. First our research design was developed to minimise common method bias, but this cannot be ruled out entirely. By using different sources, we were able to separate variance that would normally be linked to one source. As such, it was possible to reduce common method bias which might potentially inflate the different relations. We also took a number of additional precautions to minimise even further the potential common method bias. For example, we stressed to respondents that participation was completely anonymous which has been argued to reduce common method bias even further (Podsakoff, MacKenzie, Lee, & Podsakoff, 2003).

Second, the hypotheses have been explored and tested in a sample drawn from the Belgium context only. Naturally, this reduces the possibility of generalising the findings across countries and contexts. Thus, future research should consider testing the research in different contexts such as developing countries, which conditions can drastically vary from those in Europe. Additionally, this study used a cross-section survey to collect information from different sources; preferably, future research should consider using a longitudinal design in collecting both quantitative and qualitative data so as to have better view on directionality.

Third, in our study we did not hypothesize a direct effect of servant leadership on creativity and OCB. First, we considered that not necessarily servant leadership but rather its intermediate process Team-Member Exchange (TMX) would relate to employees’ creativity and both forms of OCB. As such, the intermediate variable would be a stronger predictor instead of the more distant variable, servant leadership. On an analytical level, Shrout and Bolger (2002) and Collins, Graham, & Flaherty (1998) as well as MacKinnon (2000) have argued that to find evidence for an indirect effect one does not require the independent variable to be significantly related to the dependent variable. In other words, an indirect effect is said to occur when the relationship between the independent and dependent variable becomes non-significant when entering the mediating variable (Field, 2013). Moreover, we adopted Bollen (1989) who articulated that “lack of correlation does not disprove causation” and “Correlations is neither a necessary nor a sufficient condition of causality” (p. 52). Therefore, we are in line with other scholars who adopted the same argument that “no longer imposes evidence of simple association between X and Y as a precondition” (Hayes, 2013, p. 88) (e.g. Cerin & MacKinnon, 2009; Hayes, 2009; MacKinnon, 2008; Rucker, Preacher, Tormala, & Petty, 2011; Shrout & Bolger, 2002; Zhao, Lynch, & Chen, 2010).

### Conclusion

Despite the importance of cooperation and creative solution seeking, little is documented regarding how servant leadership could help employees engage in positive exchanges among workers. This study has uncovered the importance of servant leaders by their selfless and supportive attitude, for the sake of employees’ growth and development. This study’s findings have underlined the importance of servant leadership behavior in creating a sense of ownership and high quality exchanges among employees. This in turn, encourages employees’ OCB and stimulates employees to servantly help colleagues for the sake of their welfare and that of their organisation.
